# Pathways from Food Insecurity to Health Outcomes among California University Students

**DOI:** 10.3390/nu11061419

**Published:** 2019-06-24

**Authors:** Suzanna M. Martinez, Michael A. Grandner, Aydin Nazmi, Elias Ruben Canedo, Lorrene D. Ritchie

**Affiliations:** 1 Department of Epidemiology and Biostatistics, University of California, San Francisco, 550 16th St., 2nd Floor, San Francisco, CA 94158, USA; 2 Department of Psychiatry, University of Arizona, 2800 E. Ajo Way, Tucson, AZ 85713, USA; grandner@email.arizona.edu; 3 Department of Food Science and Nutrition, California Polytechnic State University, San Luis Obispo, 1 Grand Avenue, San Luis Obispo, CA, 93407, USA; nazmi@calpoly.edu; 4 Division of Equity and Inclusion, University of California, Berkeley; 405 Sproul Hall, Berkeley, CA 94720, USA; elias_canedo@berkeley.edu; 5 Nutrition Policy Institute, University of California Division of Agriculture and Natural Resources, 2115 Milvia Street, Berkeley, CA 94704, USA; lritchie@ucanr.edu

**Keywords:** food insecurity, college students, health behaviors, sleep, diet, BMI, self-rated health

## Abstract

The prevalence of food insecurity (FI) among college students is alarmingly high, yet the impact on student health has not been well investigated. The aim of the current study was to examine the simultaneous relationships between food insecurity and health-related outcomes including body mass index (BMI) and overall health in a college student population. Randomly sampled students in the University of California 10 campus system were invited to participate in an online survey in spring 2015. The analytic sample size was 8705 graduate and undergraduate students. Data were collected on FI in the past year, daily servings of fruits and vegetables (FV), number of days in the past week of enough sleep and moderate- to vigorous-intensity physical activity (MVPA), height and weight, self-rated health, and student characteristics. Using path analysis, mediated pathways between FI, BMI, and poor health were examined through FV intake, number of days of MVPA and enough sleep. Analyses controlled for student characteristics. Mean BMI was 23.6 kg/m^2^ (SD, 5.0), and average self-rated health was good. FI was directly and indirectly related to higher BMI and poor health through three pathways. First, FI was related to fewer days of enough sleep, which in turn was related to increased BMI and poor health. Second, FI was related to fewer days of MVPA, which in turn was related to increased BMI and poor health. Third, FI was related to fewer daily servings of FV, which in turn was related to poor health. FI is associated with poor health behaviors among college students, which may contribute to higher weight status and poor health. These findings highlight the importance of food security for a healthy college experience.

## 1. Introduction

Food insecurity is defined as limited or uncertain access to nutritionally adequate and safe foods or the ability to acquire acceptable foods in socially acceptable ways due to limited financial resources [1,2] Since the Great Recession in 2007–2009, an increasing number of studies of college food insecurity have shown that students are vulnerable to food insecurity. Indeed, a recent review of U.S. college hunger reported that 40% of students experienced food insecurity based on studies conducted from 2006 and 2016 [3].

Among students at the University of California, Black and Latino students are more likely to experience food insecurity compared to their White peers. For some students, food insecurity may track from childhood into college years [4]. The stories of the psychosocial impact of food insecurity on academic performance at California’s top-tiered public universities are also empirically supported, with research showing that food insecurity is associated with poor mental health and academic outcomes [5,6,7].

Relatively little is known, however, regarding the health impact of food insecurity on college students. Several studies have shown that food insecurity is associated with elevated body mass index (BMI) in women, as well as poor health in adults [8,9,10]. In addition to nutritional deficiencies, food insecurity may promote these adverse outcomes through behavioral mechanisms [11]. For example, food insecurity can lead to a reduced intake of healthful foods (e.g., fruits and vegetables), which over time can affect health. Alternatively, food insecurity has been previously associated with poor sleep [12,13], which can also contribute to higher BMI and poor health [14]. Food insecurity may also influence BMI and health via reduced physical activity [15], which is important for maintaining overall health [16]. It is unclear whether food insecurity is related to health behaviors and health outcomes in college students. Moreover, the individual and combined relationships of diet, physical activity, and sleep to food insecurity and outcomes, including BMI and overall health, have not been simultaneously examined in any studies of adults. 

Accordingly, the present study evaluated the simultaneous relationships between food insecurity and health-related outcomes including BMI and overall health in a college student population. A path analysis approach was taken to evaluate the intermediate roles of health behaviors including diet quality (i.e., daily servings of fruits and vegetables), physical activity (i.e., moderate- to vigorous-intensity physical activity), and sleep sufficiency. The hypotheses were that (1) food insecurity is associated with higher BMI and poorer overall health, and (2) these relationships are mediated by diet quality, physical activity, and sleep sufficiency. 

## 2. Materials and Methods

### 2.1. Study Context

This study was part of the University of California (UC) Global Food Initiative, launched in 2014, to document the extent of student food insecurity on UC campuses and strategize solutions to address the issue in California colleges, nationally and worldwide (University of California and Global Food Initiative (n.d.)). In 2015 the UC statewide public university system had a student enrollment of 242,326 (34% graduate, 66% undergraduate; 29% White, 3% Black, 25% Hispanic; 65% received any financial aid support). 

#### Participants and Data Collection

This cross-sectional study used data collected from all 10 UC campuses in spring 2015. A total of 67,645 randomly sampled students were invited to participate in an online survey in one of two ways: The National College Health Assessment II (NCHA) survey (administered by the American College Health Association) [17] or an independent campus survey (administered by the UC Institutional Research and Program Planning). A total of 8932 students completed an online survey. The total 2014–2015 UC student population was compared with the 2015 study sample (weighted and unweighted) confirming that the survey sample was similar to the sampled population [4]. These comparisons and more details regarding the study design, response rate, and data collection are described in Martinez et al. [4]. Students consented electronically to participate prior to starting the survey. Participating students were entered into a lottery to be awarded prizes. Prizes included $25–$125 gift cards, computer monitors, and tablets. This incentive structure was based on previous research [18]. All research activities were approved by the Institutional Research Board at the UC Davis.

Of the 8932 total student participants, 8705 had complete data on food insecurity, 8556 had complete data on both height and weight and 8546 had complete data on self-rated health. Participants excluded from the analysis due to incomplete food security data did not differ from those included in the analysis in terms of sex and academic year (data not shown), but a greater proportion of students with missing data on food insecurity were mixed raced/other (37%), enrolled part-time (2%), or international students (14%) as compared with students with complete data on food security (11%, 0.6%, 9%, respectively; *p* < 0.05). 

### 2.2. Measures

#### 2.2.1. Independent Variable

Food insecurity assessment. Food security in the past 12 months was assessed using the validated U.S. Department of Agriculture (USDA) 6-item short form [1]. Food security scores was based on the number of affirmative answers to the six questions, using the USDA coding scheme: food secure [0–1 affirmative responses], low food secure [2–4 affirmative responses] and very low food secure [5–6 affirmative responses]. Low and very low food insecure scores were combined and coded as food insecure (1) vs. food secure (0). 

#### 2.2.2. Mediators

Three items developed for the NCHA survey were used in both online surveys to assess health behaviors, including diet quality, sleep sufficiency, and physical activity. 

Daily serving of fruits and vegetables. Students were asked ‘How many servings of fruits and vegetables do you usually have per day?’ as a measure of diet quality. The following serving information was provided to assist in self-reporting fruit and vegetable intake: 1 serving = 1 medium piece of fruit; ½ cup fresh, frozen or canned fruit/vegetable; ¾ cup fruit/vegetable juice; 1 cup salad greens or ¼ cup dried fruit. Response options, in servings/day, were 0, 1–2, 3–4, and 5 or more. Data were reverse coded; a higher number indicated fewer daily servings of fruits and vegetables (FV).

Number of days of enough sleep. Students were asked ‘On how many of the past 7 days did you do the following? Get enough sleep so that you felt rested when you woke up in the morning.’ Response options ranged from 0 days to 7 days. Number of days of enough sleep was used as a measure of sleep sufficiency, and reverse coded; a higher number of days indicated fewer days of sleep sufficiency. 

Number of days of moderate- to vigorous-physical activity. Students were asked ‘On how many of the past 7 days did you do the following? Do moderate-intensity cardio or aerobic exercise for at least 30 min; Do vigorous intensity cardio or aerobic exercise for at least 20 min; and Do 8–10 strength training exercises (such as resistance weight machines) for 8–12 repetitions each.’ Response options ranged from 0 days to 7 days. Days of moderate physical activity, vigorous physical activity and strength training were combined to represent the total number of days in MVPA, and reverse coded; a higher number indicated fewer days of MVPA.

#### 2.2.3. Dependent Variables

Body mass index. Students self-reported their height and weight; BMI was calculated as weight in kg divided by the square of height in meters (kg/m^2^).

Poor health. Students were asked ‘How would you describe your general health?*’* Response options were poor (1), fair (2), good (3), very good (4), and excellent (5). Data were reverse coded.

Control variables. A range of control variables from the survey were included in the analyses to properly specify relationships between food insecurity and student health. We included covariates that are associated with food insecurity and BMI in the literature that could in part be driving any associations found between food insecurity and health. Variables included race/ethnicity, sex, childhood history of food insecurity, being a financial aid recipient, number of hours worked for pay, being an undergraduate student, and campus affiliation (to adjust for clustering of students within campuses).

### 2.3. Analysis

Descriptive statistics were used on student characteristics. Differences by food security status (food secure vs. food insecure) were computed using independent *t*-tests for continuous variables, with significance at *p* < 0.05. Chi-square post hoc tests were performed to compare food-secure and food-insecure groups. Descriptive statistics were performed using IBM SPSS Statistics for Windows, Version 25.0 (IBM Corp, Armonk, NY, USA)

To move beyond independent associations and provide a more comprehensive understanding of food insecurity in the context of student health, we performed path analysis to simultaneously examine individual and combined relationships of diet, physical activity, and sleep to food insecurity and health. Thus, we were able to examine direct and indirect associations of food insecurity on BMI and poor health. Mediators included enough sleep, MVPA, and F/V intake. In addition, we explored partial mediation from sleep and F/V intake to BMI and poor health through MVPA. Covariates in the model included race/ethnicity, sex, childhood history of food insecurity, being a financial aid recipient, number of hours worked for pay, being undergraduate student, and campus affiliation. Modeling was performed using Mplus 7 (Muthén & Muthén, Los Angeles, CA, USA), with full information maximum likelihood to accommodate missing values. Overall model fit was determined using the following fit indices: confirmatory fit index (CFI ≥ 0.95) root mean square error of approximation (RMSEA ≤ 0.06; and standardized root mean square residual (SRMR ≤ 0.08). All paths were considered statistically significant if the *p*-value was less than 0.05. The parameter estimates, standard errors, z-statistics, and squared multiple correlations were inspected for sign and magnitude. Mediation was tested using the INDIRECT command within Mplus, which estimates indirect effects with delta method standard errors [19]. 

## 3. Results

### 3.1. Participant Characteristics

Characteristics of the sample are reported in Table 1. On average, students were 23.2 (SD, 5.8) years old; and 67% female, 34% white, 31% Asian, and 21% Hispanic. Lifestyle behaviors and health factors are reported in Table 2. Average BMI was 23.7 (4.8) kg/m^2^ and self-rated health was 2.6 (SD, 1.0), which is equivalent to good. Statistical differences were found between food-secure and food-insecure groups in mean days of enough sleep (3.6, SD, 2.0 vs. 2.9, SD, 1.9, respectively), MVPA (1.8, SD, 1.5 vs. 1.7, SD, 1.6, respectively), and daily F/V servings (2.5, SD, 1.4 vs. 2.0, SD, 1.3, respectively). A higher prevalence of students experiencing food insecurity were overweight or obese (i.e., BMI at or above 25 kg/m^2^) (33%) compared to students who were food secure (25%). 

### 3.2. Path Analysis

**Figure 1:**
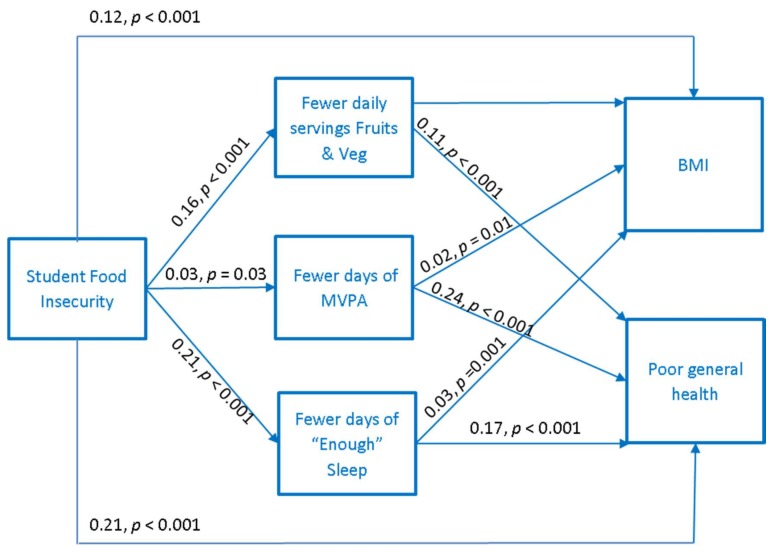
Behavioral mechanisms connecting food insecurity to body mass index (abbreviated as BMI) and poor general health in university students (*n* = 8482), controlling for race/ethnicity, sex, being a financial aid recipient, number of hours worked for pay, and an being undergraduate student, and campus affiliation. Beta coefficients are standardized. Model fit: CFI = 0.95; RMSEA = 0.04; SRMR = 0.04.

Results of the path analysis are displayed in Figure 1. The *a priori* model fit the data well (CFI = 0.95, RMSEA = 0.04, SRMR = 0.04). As hypothesized, FI was directly (β_BMI_ = 0.12, *p* < 0.001; β_health_ = 0.21, *p* < 0.001) and indirectly related to an increase in BMI and poor health through three mechanisms. First, FI was related to fewer days of enough sleep (β = 0.21, *p* < 0.001), which in turn was related to an increase in BMI (β = 0.03, *p* = 0.001) and poor health (β = 0.17, *p* < 0.001). Second, FI was related to fewer days of MVPA (β = 0.03, *p* = 0.03), which in turn was related to an increase in BMI and poor health (β = 0.23, *p* < 0.001). Third, FI was related to fewer daily servings of FV (β = 0.16, *p* < 0.001), which in turn was related to poor health (β = 0.11, *p* < 0.001). 

## 4. Discussion

The present study evaluated the relationship between food insecurity and outcomes including BMI and overall health, and the intermediate roles of diet quality (i.e., daily servings of FV), physical activity (i.e., MVPA), and sleep sufficiency. Overall, the findings showed that food insecurity was directly associated with both higher BMI and poor health, and that diet quality, physical activity, and sleep sufficiency all played intermediary roles. Thus, sleep sufficiency, diet quality, and physical activity represent important behavioral pathways linking food insecurity to BMI and overall health. 

We found that college students experiencing food insecurity were more likely to have a higher BMI, which is similar to what other studies have found among women and older adults [9,20]. This important finding highlights the perception that food insecurity contributes to lower BMI due to inadequate food access. Indeed, the food insecurity-obesity paradox may be explained by the proposed hypothesis that food insecurity may cause weight *gain* as a result of increased consumption of calorie-dense, poor quality foods, and metabolic changes due to episodic undereating [21,22]. Our findings support this hypothesis, and suggest that food insecurity is a risk factor for overweight or obesity in college students. Furthermore, we found that students experiencing food insecurity were more likely than their food-secure peers to rate their health as poor, which is consistent with findings in a small sample of 351 mainly non-Hispanic White college students from the University of Alabama [23]. The latter study, however, did not find that food insecurity was related to higher BMI, possibly a consequence of the study sample of non-Hispanic Whites, who are at lower risk for overweight and obesity than Hispanics and Blacks [23]. 

We found multiple pathways by which food insecurity may be related to BMI and health outcomes. These pathways involved lifestyle health behaviors—diet quality, sleep sufficiency, and physical activity. To our knowledge, this is the first study to examine these behavioral mechanisms, and further delineate the mechanisms by which food insecurity may affect health for students in higher education. Specifically, students experiencing food insecurity consumed fewer fruits and vegetables, which was related to worse overall health, but not to BMI. Additionally, students experiencing food insecurity reported fewer days of sleep sufficiency, which was also related to an increase in BMI and poor overall health. In a population-based study, using NHANES 2005–2010 data, men and women experiencing food insecurity were more likely to report sleep complaints compared to adults who were food secure [24]. Our finding that fruit and vegetable intake was not protective of BMI is surprising, but is consistent with a study of Arizona college freshman [25]. Furthermore, fruit and vegetable consumption was generally low in the UC sample. Walker and Kawachi have observed that when experiencing food insecurity and limited food resources, adults are more likely to consume energy-dense, nutrient-poor foods over nutrient-dense ones such as fruits and vegetables [26]. Lastly, physical activity mediated the relationship between both food insecurity and BMI and poor overall health. This finding is consistent with the study of Arizona college freshman, which found that food insecurity was associated with less healthy physical activity on campus [25]. In a study using NHANES 2003–2006 data, food insecurity was related to less physical activity and lower likelihood of meeting physical activity recommendations in children [15]. 

Taken together, these findings suggest that food insecurity may affect student health via multiple concurrent behavioral mechanisms that include poor lifestyle behaviors, which are known risk factors for poor health over the long term. For example, insufficient sleep is linked to a constellation of cardiometabolic markers (e.g., high blood pressure, high triglycerides) and chronic disease (e.g., type 2 diabetes, hypertension) [27,28,29]. The long-term longitudinal impact of food insecurity on health has yet to be determined. Indeed, in the midst of an obesity epidemic, the college years may be a vulnerable period given student risk for weight gain, commonly referred to as *the freshman 15* (pounds), which may be either exacerbated or confounded by food insecurity. 

### 4.1. University Student Context of Food Insecurity

It is reasonable to suggest that the impact of the high cost of attending a 4-year university coupled with the Great Recession has manifested in countless experiences of food insecurity in the U.S. college population [3]. For college students, tuition and fees have nearly doubled since the Great Recession. These costs do not include basic needs like food, housing and hygienic products. This burden is heightened for those living in high cost-of-living metropolitan cities. For instance, almost half of UC students are from low-income families and receive Pell Grants (federal financial aid for students from low-income families; 12 semester maximum); 42% (~88,000) are the first in their families to attend a 4-year university (referred to as first-generation) [30]. In 2016, 55% of low-income (family income <$50,000) and 56% of first-generation UC students experienced food insecurity. Although, food insecurity tracks from childhood to college-age, students without a history of childhood food insecurity are also affected [4]. Prior research of food insecurity during college [31] suggests that food insecurity is not an issue that only affects students from low-income families. 

### 4.2. Higher Education Institutional Strategies for Addressing Food Insecurity

Our results suggest that it is critical to develop institutional strategies to prevent and address food insecurity at colleges and universities. This could include integrating basic needs programs for incoming students and administering screeners for food insecurity to students entering the university system. Furthermore, outreach opportunities to the student body should be capitalized upon to promote campus food resources and assistance programs. Lastly, tracking food insecurity at the institutional level is essential. 

Establishing on-campus food pantries has been the immediate response to addressing food insecurity on U.S. college campuses. Yet, there is a need to establish longer-term solutions. On-campus food pantries are not equipped to be a constant and reliable source of nutrition for college students, as they typically function on volunteer time, donations, small budgets, limited quantities of food, and limited staffing. Moreover, food donations are not always the healthiest foods, and fresh fruits and vegetables are limited. 

In general, ensuring that students have their basic needs (food and housing) met is gaining recognition as a major challenge across public higher education. These findings can be used to support student basic needs initiatives and policies to ensure that students are meeting their nutritional needs. In addition, public university systems and policymakers should advocate for students meeting their nutritional needs, just as they prioritize tuition and fees. One feasible strategy would be advocacy regarding making it easier for students to be eligible and to apply for the U.S. Supplemental Nutrition Assistance Program, also known as SNAP. 

However, the stigma attached to food insecurity may make it harder for students to seek the assistance needed [6]. Food insecurity is socially sensitive topic; likely heightened in a socially developmental period such as young adulthood [32], and for students who have transitioned into food insecurity. Reframing SNAP for college students a could help to reduce stigma related to food stamps, and correct the misperception that SNAP negatively impacts students’ financial aid package, but rather additive to their package. Future efforts at promoting food access programs should aim to change social attitudes about users of programs such as SNAP and food pantries. Educating students about SNAP and shifting the mindset to “skills building” that includes food literacy, nutrition education, and living on a budget could help reduce the stigma attached to food insecurity [6]. 

### 4.3. Federal Level Policies Influencing Basic Needs in Higher Education

At the national level, there is a need to challenge funding for higher education and financial aid. A university school meal program where students can qualify for free, reduced price or full price meals on campus—modeled after the National School Lunch Program and School Breakfast Program in grades K-12—has also been recommended, with some private universities already providing such a service. While being a costly program to create, it would ensure equity in access to food and potentially reduce stigma.

Today, Federal Pell grant is at a four-decade purchasing power low. Financial aid systems are antiquated and student packages have not kept pace with increasing tuition and cost of living, leaving students, even those who work, in financially precarious positions at the end of academic terms [33]. Along with more aggressive efforts to decrease tuition and increase college accessibility, financial aid algorithms should be realigned with actual tuition and living expenses to decrease the acute burden on college students, even though this may translate to higher debt upon graduation. Educating students prior to entering college on personal finance, college finances, and strategies to support their basic needs (e.g., cooking skills) is essential. This is especially needed for first-generation college students, low-income to working class students, and students who will be living on their own for the first time in their lives. The issue here is the major focus on high school students (across all income spheres) taking advanced placement and standardized tests that get them into college, with little to no focus on how to thrive in college. We must significantly improve the way we prepare and support student wellness and economic security. 

### 4.4. Limitations

One important limitation of this analysis is its cross-sectional nature. No statements of causality can be made or implied. Although the path analytic approach provides a framework for inferring direction without causality, this study cannot determine the causal pathways linking food insecurity and poor health. Future longitudinal studies are needed to determine causal relationships. Another limitation is the subjective self-report nature of the measurements of food insecurity, diet, physical activity, sleep, weight, and health. Because these dimensions were not assessed objectively or prospectively, there are risks of recall bias, measurement error, and other sources of inaccuracy. Future studies should employ more rigorous methods for assessing diet (e.g., 24-h recalls or food records), physical activity (e.g., energy expenditure and accelerometry), sleep (e.g., sleep diary or actigraphy), BMI (measured height and weight), and metabolic markers (e.g., cholesterol, glucose, blood pressure). This study was also limited to students at the University of California—one of two public university systems in the state and results are likely not generalizable to all college students. Strengths of this study include the diverse sample from a large university system. This sample was similar to the true populations of students at the 10 UC campuses.

## 5. Conclusions

This study used path analysis to illustrate underlying mechanisms by which food insecurity is related to health outcomes among college students. Food insecurity was associated with higher BMI and poor health, while diet quality, physical activity, and sleep sufficiency were important mediators. Future research should examine the impact of behavioral health interventions, including SNAP and food pantry assistance, on mitigating some of the adverse effects of food insecurity in college students. Additionally, studies could examine the impact of food insecurity on health after college examinations. Lastly, institutions of higher learning need state and federal investment in higher education to ensure that students have the resources that equitably support their health and their basic needs.

## Figures and Tables

**Table 1:** Characteristics of 8705 students surveyed in spring 2015 about food insecurity at University of CA, and differences by food security status in the past 12 months; data represent % (*n*) unless otherwise specified.

Characteristics	Total Sample	Food Secure	Food Insecure
**Total**	100 (8705)	61 (5627)	40 (3438)
Age (years), mean, SD	23, 6	24, 5	23, 5
Gender			
Female	67 (5818)	67 (3514)	67 (2304)
Male	33 (2817)	33 (1720)	32 (1097)
Race/ethnicity			
Non-Hispanic White	34 (2974)	41 (2147)	24 (827)
Non-Hispanic Black	2 (206)	2 (100)	3 (106)
Hispanic	21 (1844)	15 (797)	31 (1047)
Asian	31 (2691)	31 (1637)	31 (1054)
Mixed race or other	11 (990)	11 (586)	12 (404)
Academic level			
Undergraduate	66 (5720)	57 (3006)	79 (2714)
Graduate	34 (2994)	43 (2233)	22 (711)
Hours worked for pay			
0 h	48 (4121)	49 (2557)	46 (1564)
1–19 h	31 (3662)	27 (1479)	36 (1233)
20+ h	16 (1403)	25 (1257)	19 (631)
Received financial aid, need-based scholarship, grant, loan	65 (5628)	73 (1386)	82 (1247)
Childhood history of food insecurity	22 (1926)	24 (467)	76 (1459)

*Note*: Not all students had complete data on demographic characteristics; therefore, percentages may not add up to 100 percent.

**Table 2:** Lifestyle behaviors and health factors for 8705 students surveyed in spring 2015 at University of CA; data represent mean (SD) unless otherwise specified.

	Total Sample	Food Secure	Food Insecure
**Health factors**			
Body mass index (kg/m^2^) ***	23.65 (4.82)	23.22 (4.23)	24.30 (5.55)
Poor self-report health (median, interquartile range)	3 (2–3)	2 (2–3)	3 (2–4)
Overweight/obese (%, n) ***	28, 2349	25, 1270	33, 1110
**Lifestyle behaviors**			
Daily servings of F/V ***	2.30 (1.38)	2.50 (1.41)	2.00 (1.28)
No. days of enough sleep ***	3.34 (1.99)	3.64 (1.98)	2.87 (1.91)
No. days of MVPA **	1.77 (1.56)	1.81 (1.53)	1.72 (1.60)

*Note*: Range for poor self-rated health is excellent (0) to poor (5); no. days of enough sleep and MVPA refers to the last 7 days; fruits and vegetables abbreviated as F/V. *** Independent *t*-tests between food-secure and food-insecure groups significantly different at *p* < 0.001; ** *p* < 0.01.

## Abbreviations

Food insecurity	(FI)
Body mass index	(BMI)
Fruits and vegetables	(FV)
Moderate to vigorous intensity physical activity	(MVPA)
National College Health Assessment	(NCHA)
University of California	(UC)
